# Understanding transmission risk and predicting environmental suitability for Mayaro Virus in Central and South America

**DOI:** 10.1371/journal.pntd.0011859

**Published:** 2024-01-09

**Authors:** Michael Celone, Sean Beeman, Barbara A. Han, Alexander M. Potter, David B. Pecor, Bernard Okech, Simon Pollett

**Affiliations:** 1 Department of Preventive Medicine & Biostatistics, Uniformed Services University of the Health Sciences, F. Edward Hébert School of Medicine, Bethesda, Maryland, United States of America; 2 Cary Institute of Ecosystem Studies, Millbrook, New York, United States of America; 3 One Health Branch, Walter Reed Army Institute of Research, Silver Spring, Maryland, United States of America; 4 Walter Reed Biosystematics Unit, Smithsonian Museum Support Center, Suitland, Maryland, United States of America; 5 Department of Entomology, Smithsonian Institution—National Museum of Natural History (NMNH), Washington, DC, United States of America; 6 Infectious Disease Clinical Research Program, Department of Preventive Medicine and Biostatistics, Uniformed Services University of the Health Sciences, Bethesda, Maryland, United States of America; 7 Henry M. Jackson Foundation for the Advancement of Military Medicine, Inc., Bethesda, Maryland, United States of America; McGill University Faculty of Medicine and Health Sciences, CANADA

## Abstract

Mayaro virus (MAYV) is a mosquito-borne *Alphavirus* that is widespread in South America. MAYV infection often presents with non-specific febrile symptoms but may progress to debilitating chronic arthritis or arthralgia. Despite the pandemic threat of MAYV, its true distribution remains unknown. The objective of this study was to clarify the geographic distribution of MAYV using an established risk mapping framework. This consisted of generating evidence consensus scores for MAYV presence, modeling the potential distribution of MAYV in select countries across Central and South America, and estimating the population residing in areas suitable for MAYV transmission. We compiled a georeferenced compendium of MAYV occurrence in humans, animals, and arthropods. Based on an established evidence consensus framework, we integrated multiple information sources to assess the total evidence supporting ongoing transmission of MAYV within each country in our study region. We then developed high resolution maps of the disease’s estimated distribution using a boosted regression tree approach. Models were developed using nine climatic and environmental covariates that are related to the MAYV transmission cycle. Using the output of our boosted regression tree models, we estimated the total population living in regions suitable for MAYV transmission. The evidence consensus scores revealed high or very high evidence of MAYV transmission in several countries including Brazil (especially the states of Mato Grosso and Goiás), Venezuela, Peru, Trinidad and Tobago, and French Guiana. According to the boosted regression tree models, a substantial region of South America is suitable for MAYV transmission, including north and central Brazil, French Guiana, and Suriname. Some regions (e.g., Guyana) with only moderate evidence of known transmission were identified as highly suitable for MAYV. We estimate that approximately 58.9 million people (95% CI: 21.4–100.4) in Central and South America live in areas that may be suitable for MAYV transmission, including 46.2 million people (95% CI: 17.6–68.9) in Brazil. Our results may assist in prioritizing high-risk areas for vector control, human disease surveillance and ecological studies.

## Author summary

We used a comprehensive, georeferenced compendium of Mayaro virus (MAYV) occurrence to model the suitability for MAYV in Central and South America. Our boosted regression tree model incorporated 195 MAYV occurrence locations and 9 gridded environmental covariates to generate a 5 x 5 km continuous surface of MAYV suitability across Central and South America. Using this distribution model, we estimated the total population residing in areas that are suitable for MAYV transmission. Furthermore, we developed evidence consensus scores for each country in our study region that synthesized a variety of sources to assess the overall evidence of known MAYV transmission. Our study provides a contemporary estimate of MAYV distribution using a well-established disease mapping framework. This information provides an evidence base that can guide disease surveillance (including human cases and ecological studies) and vector control efforts in Central and South America. This is especially useful in regions with high predicted MAYV suitability but only moderate evidence of current MAYV transmission (e.g., Guyana and Suriname).

## Introduction

Mayaro virus (MAYV) is a mosquito-borne *Alphavirus* that was first detected in Trinidad in 1954 [1]. MAYV has caused periodic outbreaks throughout Latin America [2] and serological surveys and syndromic surveillance studies suggest widespread circulation in the region [3]. Some researchers have hypothesized that MAYV has broader epidemic potential and raised alarm about its apparent increased geographic spread [4, 5].

MAYV can cause debilitating arthralgia or arthritis that can persist for months after initial infection [6]. However, MAYV often results in non-specific febrile symptoms that are similar to other vector borne diseases such as dengue or Zika [7]. Therefore, clinical diagnosis is often difficult and accurate estimates of disease burden remain elusive. This is further complicated by the many limitations of serological diagnostics including the cross-reactivity of antigenically similar viruses [8]. Supportive care remains the current standard of clinical treatment for MAYV as no licensed vaccine or antiviral treatment currently exists.

Limited studies on MAYV ecology suggest that this virus is maintained in a sylvatic transmission cycle involving arboreal mosquito vectors and non-human animal reservoirs. High seroprevalence among non-human primates (NHPs) [9] suggests they may be involved in the MAYV transmission cycle, although their precise role is inconclusive. In addition, MAYV antibodies have been detected in other mammals including rodents and marsupials [10]. Risk factors, such as living in close proximity to forested areas [11] and hunting in the rainforest [12] have been linked to MAYV infection in humans. This underscores the significance of the sylvatic transmission cycle and the potential for spillover events. However, MAYV occurrence in urban environments such as Manaus, Brazil has led to concerns about an urban transmission cycle driven by *Aedes aegypti* mosquitoes [13]. Though studies of wild-caught mosquito populations implicated the canopy-dwelling *Haemagogus (Hag*.*) janthinomys* mosquito as the primary vector during a major outbreak in Brazil [9], *Aedes aegypti* and *Aedes albopictus* have demonstrated the potential to transmit MAYV in laboratory settings [14].

A prior epidemiological alert by the Pan American Health Association (PAHO) stressed the importance of heightened awareness and expanded surveillance of MAYV in Central and South America [15]. Ideally, MAYV spillover and outbreak prevention would be guided by granular maps of MAYV risk enabling targeted febrile surveillance and ecological surveillance efforts and better tailored risk communications to endemic populations and travelers. However, the precise areas of risk from MAYV remain unclear due to limited data on MAYV occurrence [16], underscoring a fundamental need for a more comprehensive and georeferenced dataset on MAYV occurrence.

Here, we provide a significant update to the current state of knowledge on MAYV transmission risk across Central and South America. We adopted a well-established machine-learning based disease mapping approach originally developed by ecologists to model species distributions but has since been successfully applied to several medically-relevant vector-borne pathogens [17–19]. These methods are particularly powerful for leveraging biological and ecological information underpinning a sylvatic disease system to generate biologically realistic and spatially explicit predictions when epidemiological data are still sparse. Many of these models rely on machine learning techniques including boosted regression trees (BRT) [20] to develop a multivariate relationship between disease occurrence locations and relevant climatic or environmental covariates that impact disease transmission.

In order to develop a contemporary estimate of MAYV risk in Central and South America, we applied a predictive mapping approach with three components: (1) scoring the total evidence supporting ongoing MAYV transmission within each country (i.e., evidence consensus scores); (2) modeling the likely distribution of MAYV suitability throughout Central and South America; (3) estimating the total population residing in areas with a high suitability for MAYV transmission. Compared to previous estimates, these updated datasets and analyses suggest that MAYV poses a substantial and possibly underestimated threat to Central and South America.

## Methods

### Evidence consensus

Collating published reports of MAYV is an important first step in clarifying its distribution. However, heterogeneous surveillance across countries and incomplete or unclear reporting of epidemiological data may impact our ability to definitively say that MAYV is present in a certain location. An evidence consensus approach takes several information sources into account in order to score the total available evidence supporting the presence or absence of a disease in a given country. This approach has been used previously to provide a refined description of the spatial limits of several pathogens including dengue [21], leishmaniasis [22], podoconiosis [23], and Lassa fever [24]. These studies considered multiple data sources to develop an evidence consensus score for disease presence or absence, including health organization status, peer reviewed evidence, case data, animal infection, economic status, and other supplementary evidence. We followed a similar procedure to generate a consensus score for each country in Latin America that quantifies the evidence supporting MAYV presence. This score ranged from 0 (“No evidence of MAYV presence”) to 21 (“Complete evidence of MAYV presence”) based on the categories described below (see Table 1 for a summary of categories and possible scores).

**Table 1:** Evidence Categories and Possible Scores.

Evidence Category	Score
Health organization status	
Both GIDEON and PAHO/WHO	3
Either GIDEON or PAHO/WHO	1
Peer reviewed evidence	
Date of MAYV human occurrence	
2011–2021	3
2000–2010	2
1999 and earlier	1
Diagnostic procedure	
PCR or viral culture or PRNT	3
Serological methods (not including PRNT)	2
Presumptive diagnosis or not specified	1
Outbreaks and clinical cases	
20+ cases from 2011–2021	6
20+ cases from 2000–2010	5
20+ cases 1999 and earlier	4
<20 cases from 2011–2021	3
<20 cases from 2000–2010	2
<20 cases 1999 and earlier	1
If no case data: health expenditure in 2017 and adjacency	
HE <100 USD and 2 or more neighbors	6
100 USD ≤ HE <500 USD and 2 or more neighbors	5
HE ≥500 USD and 2 or more neighbors	4
HE <100 USD and 1 neighbor	3
100 USD ≤ HE <500 USD and 1 neighbor	2
HE ≥500 USD and 1 neighbor	1
Animal data	
Infected animal from 2011–2021	3
Infected animal from 2000–2010	2
Infected animal 1999 and earlier	1
Arthropod data	
Positive arthropod from 2011–2021	3
Positive arthropod from 2000–2010	2
Positive arthropod 1999 and earlier	1
**Possible Evidence Consensus Score Categories (Maximum Possible Score: 21)**	
**Very high evidence of MAYV presence**	**16–21**
**High evidence of MAYV presence**	**11–15**
**Moderate evidence of MAYV presence**	**6–10**
**Little to no evidence of MAYV presence**	**0–5**

#### Health organization reports (max 3 points)

International health organizations have been used previously to support evidence of pathogen presence or absence in specific countries [21]. We followed a similar procedure, using health reports from two sources: Pan American Health Organization (PAHO)/World Health Organization (WHO) and the Global Infectious Diseases and Epidemiology Online Network (GIDEON). The PAHO issues weekly epidemiological alerts to update the public on the occurrence of significant health events. Similarly, the WHO issues Disease Outbreak News (DONs) related to public health issues of international importance. We searched these PAHO/WHO bulletins for relevant alerts related to MAYV in a given country. Countries were assigned a score of 1 if WHO/PAHO had issued an epidemiological alert for MAYV in that country.

GIDEON is a web application that compiles relevant news on infectious disease outbreaks and designates countries as endemic/potentially endemic for each pathogen. If a country was listed as endemic/potentially endemic for MAYV in the GIDEON database, it was assigned a score of 1. If a country fulfilled both criteria (i.e., listed as endemic in GIDEON and a relevant PAHO/WHO health alert) it was assigned a score of 3.

#### Peer-reviewed evidence of human infection (max 6 points)

Based on methods proposed by Brady et al., [21] peer-reviewed evidence of human infection was scored based on the following two categories: contemporariness (3 for 2011–2021, 2 for 2000–2010, and 1 for 1999 and earlier) and diagnostic accuracy (3 for PCR, viral culture, or PRNT, 2 for other serological methods only, 1 for presumed cases without diagnostic test or cases with unspecified diagnostic test). In the case of multiple MAYV reports in a given country, the highest scoring report was used. For example, if one study in Brazil reported serological evidence of MAYV transmission (score of 2) in 1990 (score of 1) and another study in Brazil reported MAYV viral culture (score of 3) in 2019 (score of 3), Brazil would receive a score of 6 for this category. We also considered returning traveler reports for this category if the case was definitively linked to the country of travel. These reports are useful for establishing evidence of pathogen presence because diagnosis may be done more rigorously for travelers upon returning to their country of origin [21]. Data was compiled in a systematic review that was previously described [25].

#### Outbreaks and clinical cases (max 6 points)

Reported outbreaks of MAYV or clinical cases that were detected using PCR were scored according to total case numbers and contemporariness. Previous studies have used only the occurrence of reported outbreaks (with no consideration to clinical cases) to assign a score for this category [21]. However, because only a limited number of MAYV outbreaks have occurred and because diagnostic techniques have been inconsistent across these outbreaks, we also considered clinical cases diagnosed by PCR or viral culture that were not necessarily considered to be an outbreak. The scoring system for this category was adapted based on methods used by Mylne et al., [24] where higher scores were assigned to contemporary reports with 20 or more cases. In order to calculate a score for each country, we summed the cases detected across multiple studies within a single time period. For example, if two separate reports from Peru each detected 10 cases of MAYV using PCR between 2011 and 2021, we summed the reported cases (20 total) and assigned Peru 6 points for this category. However, cases from multiple reports were not summed across different time periods. Data was compiled in a systematic review that was previously described [25].

A lack of MAYV case reports in a given country is not necessarily indicative of a lack of virus transmission. It is likely that MAYV cases may go undetected due to variable surveillance or diagnostic capacity. We attempted to account for this uncertainty using healthcare expenditure (HE) as a proxy of a country’s capacity for detecting MAYV occurrence. If no outbreaks or clinical cases were reported in a given country, we used the current HE per capita from the World Health Organization 2017 dataset. Total HE for each country was designated as low (HE < $100), medium ($100 ≤ HE < $500), or high (HE ≥ $500) according to methods that were previously described [21]. We also considered a country’s proximity to other countries that have reported outbreaks or clinical cases diagnosed by PCR [24]. Adjacency to countries with outbreaks or clinical cases was combined with HE to assign a score. The highest score was assigned to countries with MAYV outbreaks/clinical cases in two or more neighboring countries and a low HE.

#### Animal and arthropod data (max 6 points)

Detection of MAYV occurrence in non-human animal or arthropod species provides additional evidence of MAYV presence. This may be indicative of the potential for disease spillover into the human population. Previous studies have considered the presence of competent vector species when calculating the evidence consensus score [19, 26]. However, because of uncertainties regarding the role of various mosquitoes in the MAYV transmission cycle (e.g., the possible role of *Aedes aegypti* in urban transmission [13]), we considered reports of any wild-caught arthropods that were identified as MAYV positive. Similarly, we assigned a separate score based on reports of infection in potential animal reservoirs. The highest scores for both animal and arthropod MAYV positivity were assigned to more contemporary studies. In the case of multiple MAYV reports in a given country, the highest scoring report was used. All data on MAYV positivity in non-human animals and arthropods was compiled in a systematic review that was previously described [27].

In order to provide more granular data throughout Brazil, we also calculated evidence scores by state (i.e., first-administrative division). Because health organization status was not available for each state, we assigned a baseline score of one to each state, and then calculated the remaining categories according to the methods described above.

### Occurrence records

We previously developed and published a georeferenced compendium of MAYV occurrence [25] based on methods that have been established for other pathogens including dengue and leishmaniasis [28–31]. MAYV occurrence among humans, non-human animals, and arthropods was compiled through a systematic review of the literature, including an evaluation of the quality of such evidence. These methods were described previously in greater detail [25, 27]. All occurrences were assigned to a point or polygon location, depending on the spatial resolution provided by the authors. Point data comprised precise locations with less than 5km of uncertainty (e.g., specific coordinates or a small town) while polygon data comprised larger areas or administrative units that exceeded 5km of uncertainty. The coordinates of point locations and polygon centroids were recorded in our database and used as the presence records in our current modeling study.

Presence points with ≤75km of uncertainty were included in our current analysis, although the majority of points had substantially less uncertainty. Following a previously published modeling study that accepted up to 65km of uncertainty [32], we chose to accept a greater level of uncertainty in our occurrence data in order to include more occurrence locations during the model development process. Due to the limited size of our occurrence dataset, we deemed that the extra information gained from each occurrence record outweighed any issues associated with the greater uncertainty of some occurrence records.

The final MAYV database contained 262 unique georeferences in 15 countries, published between 1954 and 2022. This data is available in the Dryad data repository [33]. One hundred and ninety-five of these occurrence points fell within our study region and met the ≤75km uncertainty threshold, and thus were eligible for inclusion in our model. We used the spThin package in the R statistical software to reduce clustering of presence records [34]. A 5km distance threshold was applied to ensure that no more than one presence point occurred within each pixel of our covariate layers.

### Description of covariates

We considered 10 ecologically relevant gridded variables (i.e., raster data) for inclusion in our model. These variables included various measures of topography, climate, land cover and vegetation that likely influence the MAYV transmission cycle and the distribution of MAYV risk throughout the region. Several variables were derived from NASA’s Moderate Resolution Imaging Spectroradiometer (MODIS) remote sensing platform [35]. These MODIS variables (plus an additional rainfall variable) were provided by the Malaria Atlas Project (https://malariaatlas.org/) after a gap-filling algorithm was used to account for cloud cover [36]. The variables were transformed to ensure matching spatial resolution of 2.5 arc-minutes (~5km) and matching extent. Values for the variables were extracted at each presence/pseudoabsence location and used in the modelling procedure described below.

Temperature and rainfall play an important role in vector abundance and activity [37]. Entomological surveys have demonstrated an association between *Hg*. *janthinomys* abundance and temperature [38, 39] and Alencar et al. reported that the mosquito’s presence was correlated with high temperatures ranging from 24°C–30°C [40]. Several studies have also demonstrated that large diurnal temperature range can impact larval development time, adult survival, and reproductive output in *Aedes* and *Anopheles* populations [41–43] and an ecological niche model demonstrated that mean diurnal range was one of the most important predictors of *Hg*. *janthinomys* distribution [44]. Humidity and rainfall have also been shown to impact the density of adult *Hg*. *janthinomys* mosquito populations [38, 45–48] and *Hg*. *janthinomys* biting activity was shown to peak during intense rainfall in January [46]. Due to the impact of temperature and rainfall on vector abundance and vectorial capacity, we included three climate variables in our model, namely night-time and daytime land surface temperature (LST) and cumulative rainfall. LST Night and LST Day are remotely sensed variables from the NASA MODIS MOD11A2 satellite [49]. Annual LST Day and LST Night raster layers spanning the years 2000–2020 were used to calculate a single layer representing the mean values over this time period. We also used rainfall data from the Climate Hazards Group InfraRed Precipitation with Station (CHIRPS) [50], a quasi-global data set that incorporates satellite imagery at 0.05° resolution and meteorological station data to construct gridded rainfall estimates.

In addition, *Hg*. *janthinomys* mosquitoes thrive in arboreal habitats (e.g., tropical rainforests) and oviposit in water-filled natural plant cavities (e.g., tree holes or broken bamboo) [51]. Adult mosquitoes have predominantly been found in the forest canopy at heights of 16m and 30m [38]. Therefore, the density of vegetation canopy and moisture supply may influence *Hg*. *janthinomys* abundance in a given area. To account for these factors, we included three covariates related to vegetation and surface moisture: enhanced vegetation index (EVI), tasseled cap wetness (TCW) and tasseled cap brightness (TCB). The EVI is a measure of vegetation canopy greenness displayed at a 500m spatial resolution [52] and is derived from the MODIS MCD43B4 product [53]. The EVI has been used as a covariate in previous environmental suitability models of arboviruses including Yellow Fever [54], chikungunya virus [19], and Zika virus [18]. TCW and TCB, measures of surface moisture that are used to assess land cover change, were also generated from the MODIS MCD43B4 product [55].

Previous outbreaks of MAYV have occurred in towns close to the rainforest or jungle outposts in close proximity to the forest edge [2, 12, 56]. MAYV most likely circulates in a sylvatic cycle involving canopy-dwelling mosquitoes and non-human primates, with occasional spill-overs into humans living close to the forest [9]. Entomological surveys have demonstrated that *Hg*. *janthinomys* are predominantly found in forest canopies at heights of 16m and 30m [38]. Due to the strong impact of land cover on the probability of MAYV occurrence in a given area, we included two land cover covariates, namely evergreen broadleaf forest and urban/built-up, from the MODIS MOD13Q1 product [57, 58]. These covariates represent the proportion of each raster grid cell (ranging from 0–100) that is covered by the land cover class in question, whereby a value of zero represents the absence of land cover and 100 represents complete coverage. Lastly, we included slope and elevation covariates that we accessed from the US Geological Survey’s Global Multi-resolution Terrain Elevation Data (GMTED) [59]. Elevation represents the meters above sea level at a given location. Slope values range from 0 (flat) to 90 degrees (vertical) and represent the angle of the downward sloping terrain.

**Fig 1:**
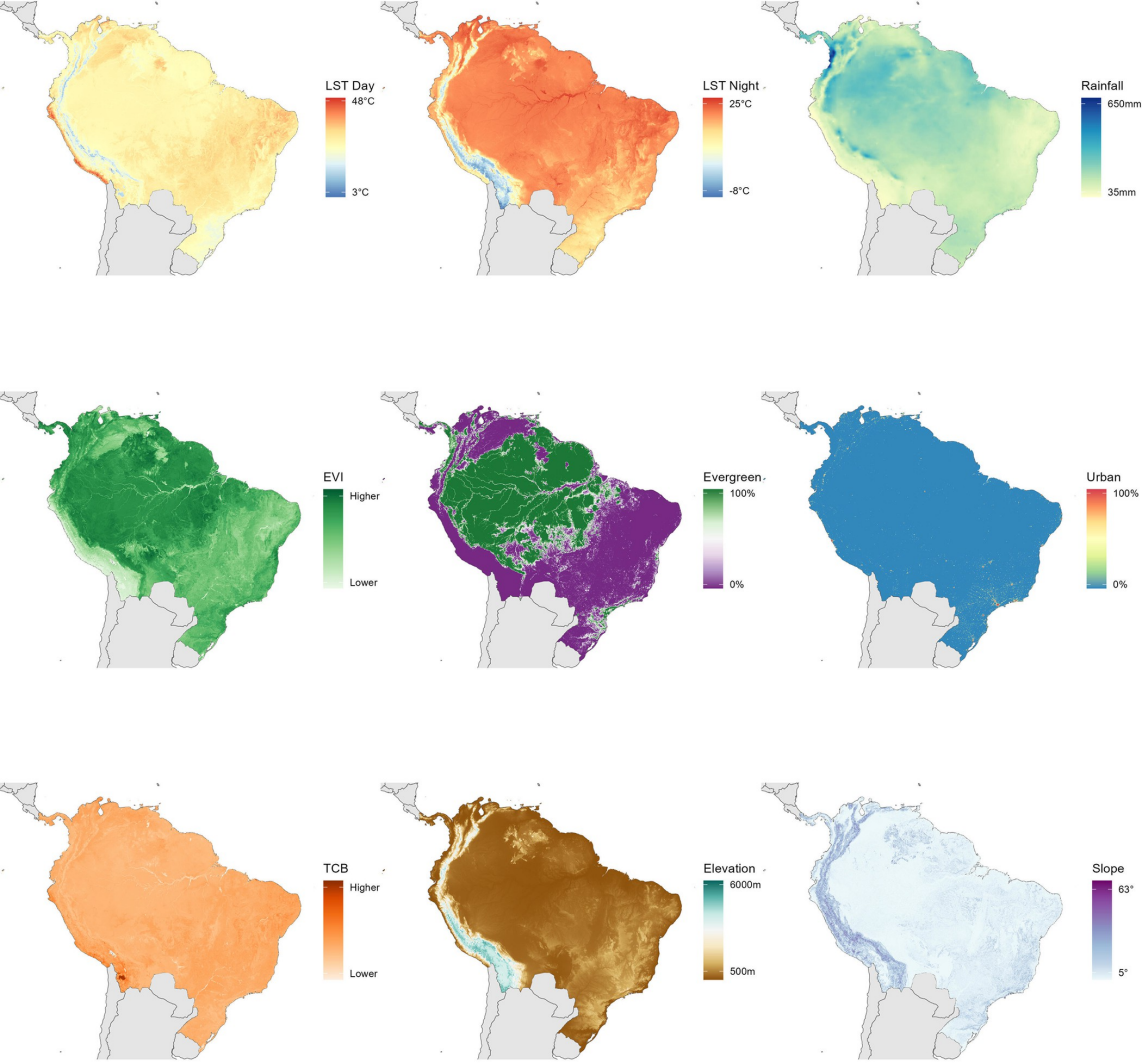
Covariates used to model the environmental suitability of MAYV. A. and B. Land surface temperature (LST) night and LST day, respectively; C. Rainfall; D. Enhanced vegetation index (EVI); E. Evergreen forest; F. Urban/built-up land cover; G. Tasseled cap brightness (TCB); H. Elevation; I. Slope. Maps were created in R using shape files from the Natural Earth public domain repository (http://www.naturalearthdata.com/).

We implemented a data-driven variable selection process in the R package SDMtune to identify variables for inclusion in our models. This process involves removal of highly correlated variables based on an algorithm that first ranks the variables by permutation importance and evaluates the correlation between the most important variable and the remaining variables. A leave-one-out Jack-knife test is then used to remove the variable that has the smallest impact on model performance according to the AUC. Based on this analysis, we removed TCW from the final model. Maps of the variables included in the model are presented in Fig 1.

### Mapping environmental suitability for MAYV occurrence

Environmental suitability models are common in the epidemiological literature to model human disease risk. A robust disease mapping framework has been established in the last decade to model the environmental suitability for medically-relevant pathogens including dengue virus [17], Chikungunya virus [19] and Zika virus [18], among others.

We used BRT to model the environmental suitability for MAYV occurrence across our study region. This algorithm uses regression or classification trees to partition the dataset using recursive binary splits. It also incorporates boosting into the model-building process, a procedure that combines many simple models to improve overall model accuracy. The boosting algorithm is an iterative process that fits many small trees sequentially, building on previously fitted trees to improve model performance [20]. This process incorporates a level of stochasticity by randomly selecting a subset of the data to fit each tree, thereby reducing the model variance [20]. BRT have several advantages including their ability to fit complex nonlinear relationships, handle missing data, and to accommodate many different types of covariates without any need for data transformation [20].

One of the most important aspects of modeling species distributions with presence-only data is the selection of pseudo-absence points that represent the range of environmental conditions where the species or pathogen was not detected. Random selection of pseudo-absence points may not be appropriate if the presence locations are spatially biased [60]. In most cases, the detection of disease presence locations may be subject to sampling bias if some locations are more likely to be surveyed than others (e.g., locations that are closer to roads) [61]. Therefore, pseudo-absence points should be selected with a similar level of bias as the presence points to ensure that background and presence locations are biased in the same manner [61]. Following the methods of previous modeling studies [24, 62–64], we selected 10,000 background points from the study region, biased towards more populous areas. Therefore, population density was used as a proxy for sampling bias. Pseudoabsence points were selected using the 2° method proposed by Barbet-Massin (2012) [65], whereby each pseudo-absence point was at least 2° away from a presence location. In order to improve the model’s discrimination capacity, the pseudoabsence points were down-weighted to ensure that the weighted sum of presence records equaled the sum of weighted background points [65].

We subsequently fitted 100 BRT ‘sub-models’ to separate bootstraps of the dataset. The bootstrapped datasets were chosen with replacement, subject to the condition that a minimum of 25 presence and 25 pseudo-absence points were selected. This bootstrapping procedure allowed us to quantify the uncertainty across models and to increase the model’s robustness [66]. Each sub-model was fit in R using the gbm.step procedure in the dismo package [67]. This function uses cross-validation to identify the optimal number of trees for each sub-model to improve predictive capacity. The remaining BRT hyperparameters were held at their default values (tree complexity = 4, learning rate = 0.005, bag fraction = 0.75, cross-validation folds = 10, step size = 10). The final prediction map represents the mean MAYV suitability of each 5 x 5 km pixel across our ensemble of 100 models along with the lower 2.5^th^ percentile and 97.5^th^ percentile predictions. We also generated a map of the model uncertainty, represented by per-pixel standard deviation. In order to avoid extrapolating the model far outside of regions of known MAYV transmission, the model predictions were restricted to the following countries in Central and South America with moderate, high, or very high evidence of MAYV transmission based on evidence consensus scores: Panama, Ecuador, Brazil, Colombia, Peru, Venezuela, Trinidad and Tobago, Suriname, Guyana, French Guiana and Bolivia.

The predictive accuracy of the model was assessed using the area under the receiver operator characteristic curve (AUC), sensitivity, specificity, Kappa statistic, and percent correctly classified (PCC). Metrics were calculated for each sub-model using 10-fold cross validation. The cross-validation procedure involved randomly splitting each bootstrapped dataset into 10 folds with approximately the same number of presence and absence records in each fold. The model was subsequently trained on nine of the folds and the withheld fold was used to evaluate the model performance. The performance metrics for each sub-model represent the mean values across the 10 folds. These values were then averaged across each of the sub-models to generate an estimate of overall model performance. We also calculated relative importance scores for each of the covariates. Relative importance was defined according to the relative percent contribution, which quantifies how often the model selects a variable for splitting. The scores are weighted by the squared improvement to model performance and averaged across all trees [20].

### Total population living in areas with high predicted MAYV suitability

We estimated the total human population living in areas of high predicted MAYV suitability. We first transformed the mean prediction map into a binary risk map using a previously established protocol [18], whereby a suitability threshold value was chosen that encompassed 90% of the MAYV occurrence points. Each 5 x 5 km pixel was classified as 1 (i.e., suitable) if its predicted suitability exceeded the threshold value; otherwise, it was classified as 0 (i.e., not suitable). We then determined the total population residing in suitable areas by multiplying the population count within each grid cell by the binary suitability classification and summing these values across each country. We used population count data from the Gridded Population of the World (GPW) version 4 [68]. Furthermore, we calculated uncertainty in these population estimates (i.e., 95% confidence intervals) using the 2.5% (lower) and 97.5% (upper) bounds of the BRT model prediction. Following Deribe et al. [69], we also conducted a sensitivity analysis to assess the impact of alternative suitability thresholds on the estimated population residing in suitable transmission zones.

### Dryad DOI

https://doi.org/10.5061/dryad.cfxpnvx8n [33]

## Results

**Fig 2:**
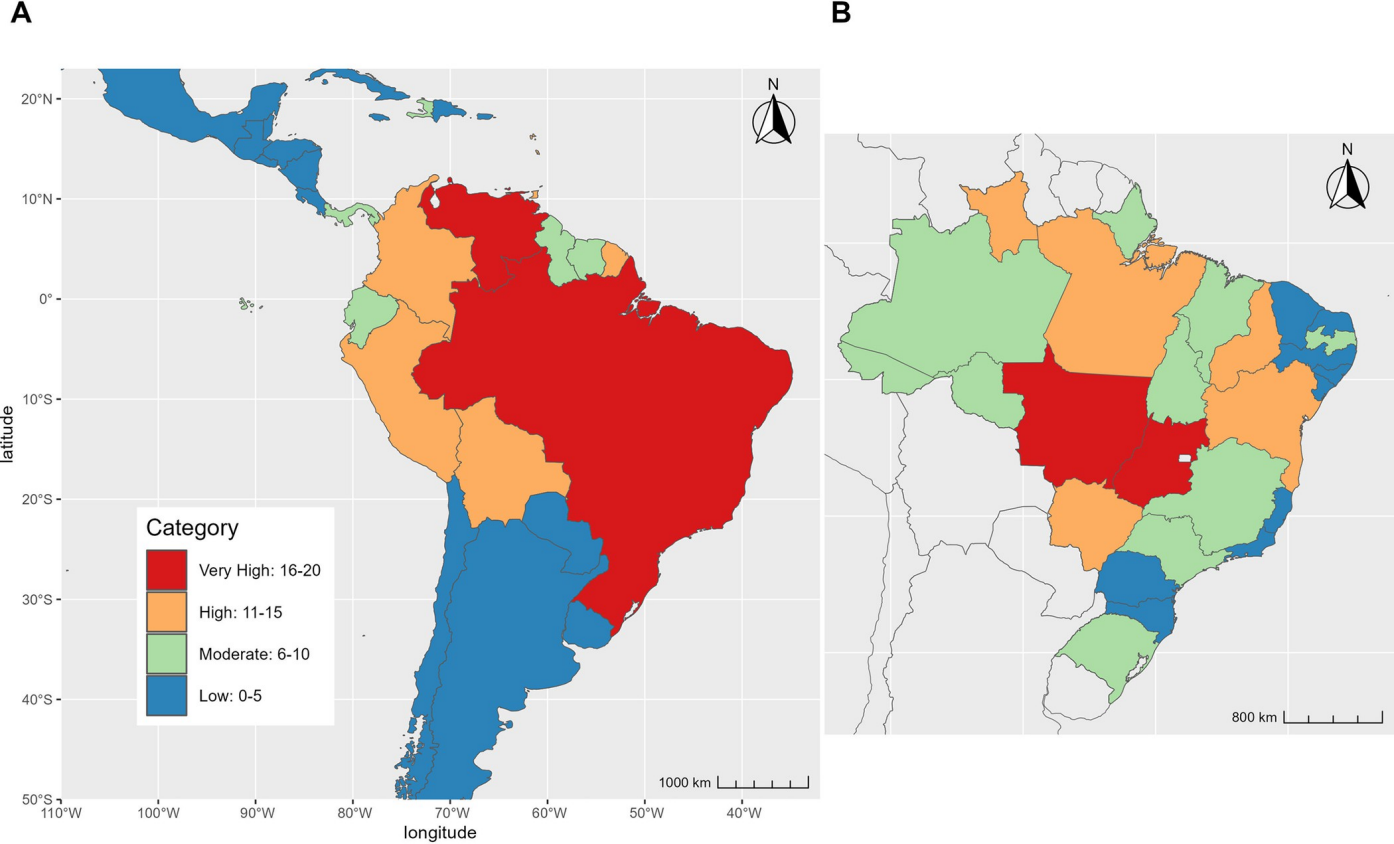
Evidence consensus scores. Evidence consensus is presented at the country level for all countries in the study (Fig 2A) and at the first-level administrative division (Fig 2B) for Brazil. Scores are based on health organization status, date of most recent MAYV occurrence; validity of MAYV diagnostic test, recency of MAYV outbreaks or clinical cases, and recency of MAYV occurrence in animals or arthropods. Blue represents very low evidence consensus while red represents very high evidence consensus. Maps were created in R using shape files from the Natural Earth public domain repository (http://www.naturalearthdata.com/).

The map of evidence consensus is presented in Fig 2A and the evidence score for each country is presented by category in the S1 and S2 Tables. Evidence consensus scores ranged from 0 (no evidence of MAYV transmission) to 19 (very high evidence of transmission). We recorded a very high evidence consensus score for Brazil and Venezuela, with scores of 19 and 16, respectively. Other countries with a high evidence consensus score included Peru (Evidence Consensus = 15), French Guiana and Trinidad and Tobago (Evidence Consensus = 13 for both), and Colombia and Bolivia (Evidence Consensus = 11 for both). We recorded evidence consensus scores ranging from very low to moderate in all Central American and Caribbean countries. Among these countries with low to moderate risk, the highest evidence consensus scores were documented for Haiti (Evidence Consensus = 10) and Panama (Evidence Consensus = 9).

Evidence consensus scores for Brazilian first-level administrative divisions are presented in Fig 2B. Evidence of MAYV transmission was highest in the central Brazilian states of Mato Grosso and Goiás, both with very high scores of 16. High evidence of MAYV was also documented in five Northern and Central states, including Pará and Bahia (Evidence Consensus = 14 for both), Roraima (Evidence Consensus = 13), Piauí (Evidence Consensus = 12), and Mato Grosso do Sul (Evidence Consensus = 11).

**Fig 3:**
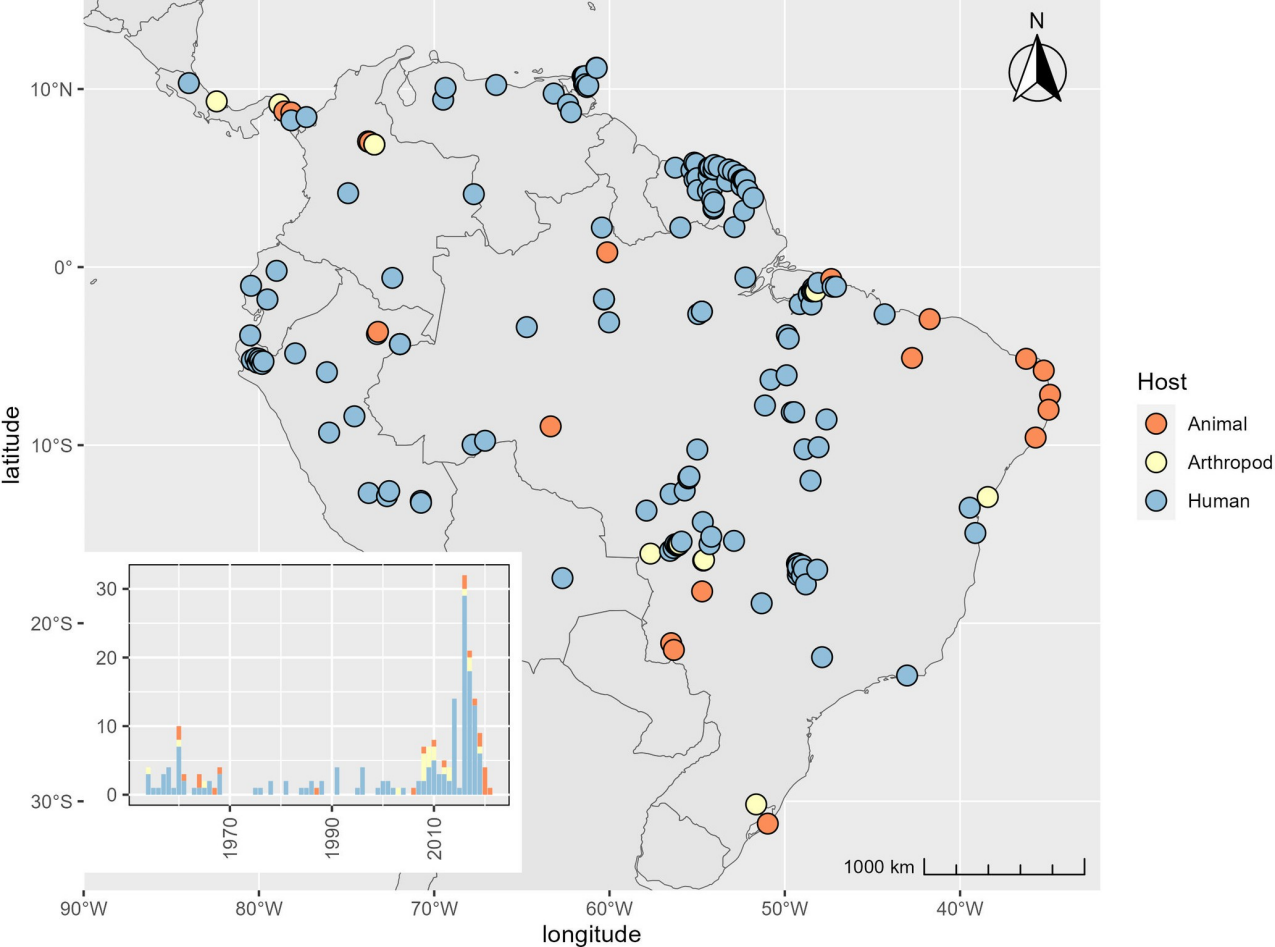
Geographic distribution and temporal trend of MAYV occurrence. The map shows the distribution of the 195 occurrence locations (before the spatial thinning procedure) that were used to construct the boosted regression tree (BRT) model. The color corresponds to the host type of each point (human, animal, or arthropod). The inset chart displays total occurrences that were reported in each year since the initial human case was detected in 1954. The map was created in R using shape files from the Natural Earth public domain repository (http://www.naturalearthdata.com/).

Fig 3 displays the 195 MAYV occurrences (human, animal, and arthropod) that were used to fit our model. The occurrence locations fell in 10 countries, most frequently in Brazil (n = 101), French Guiana (n = 25) and Peru (n = 21). MAYV occurrences were reported between the years 1954 and 2021, with the majority of cases (n = 133) occurring since the year 2000. One hundred fifty-two (78%) of the occurrence locations were detected in humans while 43 (22%) were detected in non-human animals or arthropods.

**Fig 4:**
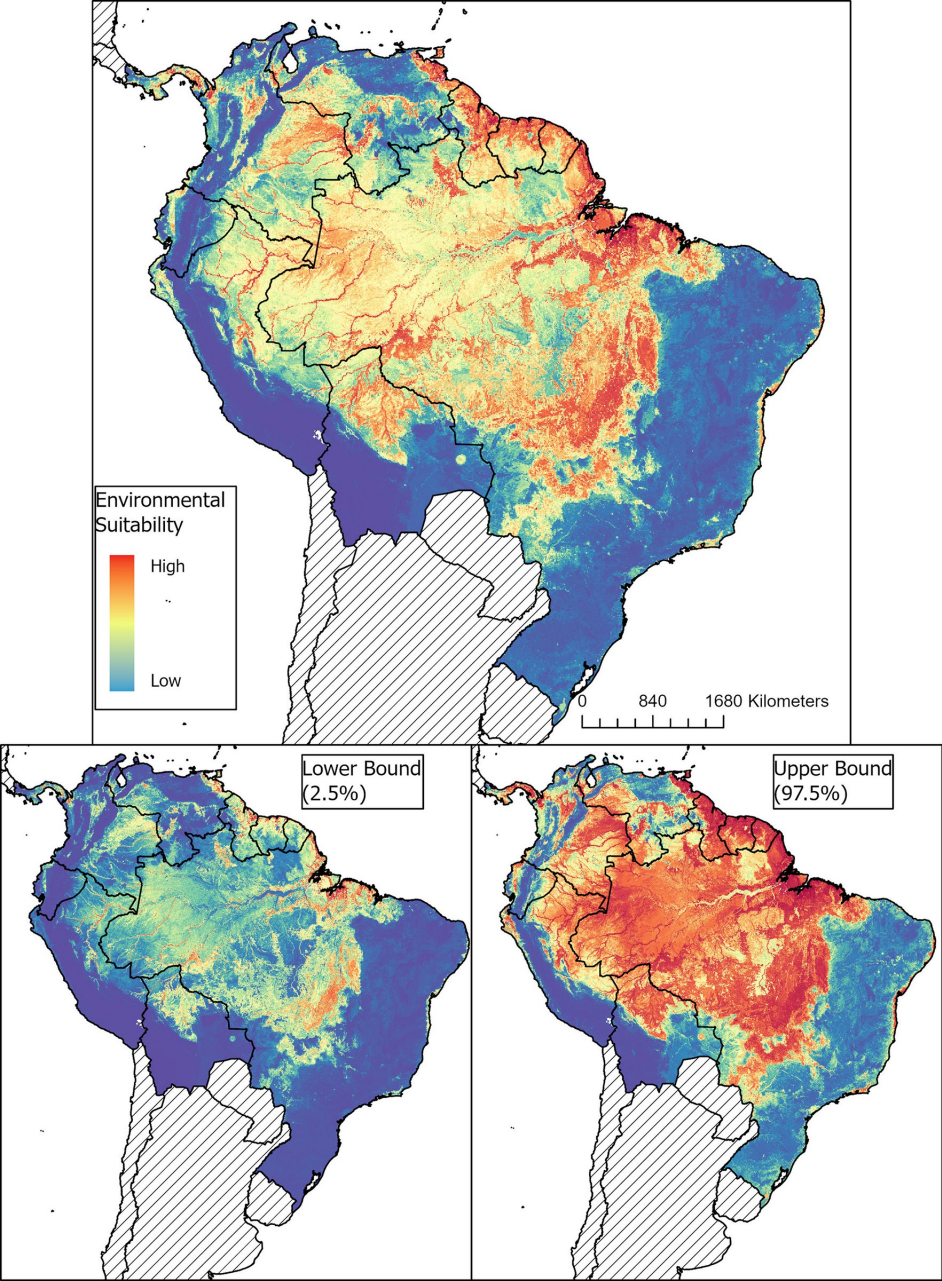
Map of environmental suitability and prediction uncertainty for MAYV occurrence. Suitability ranged from blue (0—no suitability) to red (1—very high suitability). We obtained the lower and upper bound of MAYV presence limits by fitting an ensemble of 100 BRT submodels. The base map was sourced from Global Administrative Areas (GADM) version 4.0: https://gadm.org/download_country.html.

Maps of the predicted distribution of MAYV environmental suitability, along with the lower (2.5%) and upper (97.5%) of the prediction limits, are presented in Fig 4. This risk map represents the average output across the 100 BRT sub-models. The map of model uncertainty (i.e., the per-pixel standard deviation across the 100 model runs) is presented in the S1 Fig High suitability for MAYV transmission was evident across the Amazon rainforest ecoregion in South America. The model predicted very high suitability for MAYV across a large portion of Central and Northern Brazil, especially the states of Amazonas, Acre, Pará, and Tocantins. High suitability was also predicted throughout French Guiana, Guyana, Suriname, and Trinidad and Tobago, as well as the southern portion of Colombia and Venezuela, and the north-eastern region of Peru and northern region of Bolivia. The southern region of Panama was also found to be highly suitable for MAYV transmission. After applying pairwise distance sampling to remove spatial sorting bias, the model demonstrated good predictive power with an AUC of 0.83. Other statistics from the 10-fold cross-validation procedure included the following: PCC = 87%, sensitivity = 0.82, specificity = 0.93, and Kappa = 0.75.

**Fig 5:**
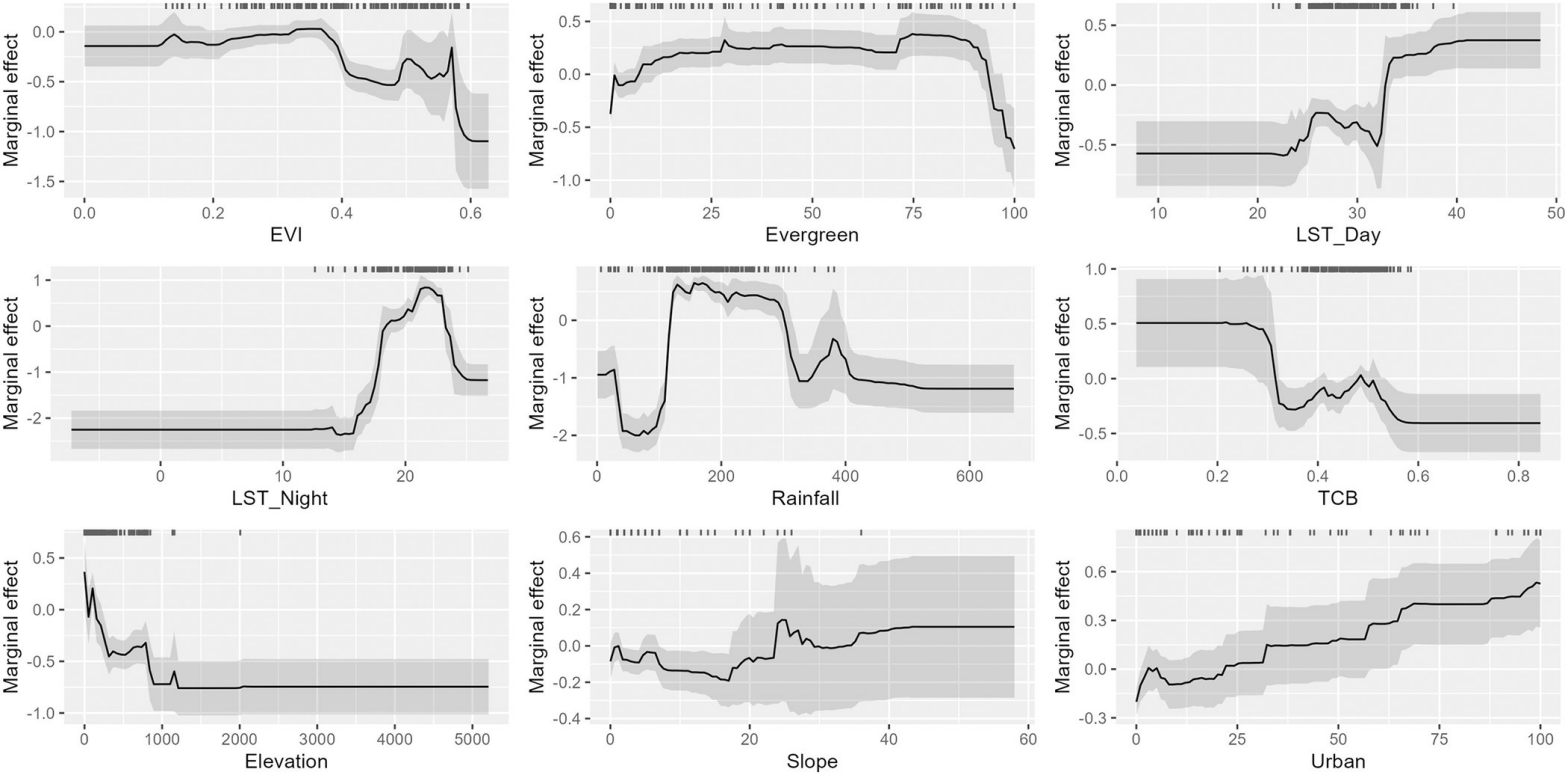
Partial dependence plots of the included variables. The solid black line represents the average response over 100 sub-models and the gray region represents one standard deviation. Tick marks represent values of each variable at occurrence locations. The y-axis represents the untransformed logit response and x-axis represents the full range of values for each covariate.

Our models showed MAYV suitability to be especially influenced by climatic variables including rainfall, with a relative importance of 34.5 (95% CI: 28.3–41.6) and nighttime LST, with a relative importance of 29.6 (95% CI: 24.4–34.0). Relative percent contributions for the remaining variables were 9.0 for elevation (95% CI: 6.2–12.8), 6.9 for EVI (95% CI: 5.1–8.9), 5.9 for urban land cover (95% CI: 4.4–7.6), 4.9 for daytime LST (95% CI: 3.8–6.5), 4.0 for evergreen land cover (95% CI: 2.5–5.7), 3.3 for TCB (95% CI: 2.1–4.9) and 1.9 for slope (95% CI: 0.7–3.5). The partial dependence plots for the covariates are presented in Fig 5. The partial dependence plot for nighttime LST reveals a steep increase in MAYV suitability around ~15°C that peaks at ~22°C and then falls. The plot for rainfall reveals a similarly steep increase starting at ~100mm that plateaus and then decreases around ~300mm, with only a minor peak at ~375mm.

We identified 0.53 as the threshold suitability value that encompassed 90% of the MAYV occurrence locations. We applied this conservative value to classify pixels as either suitable or unsuitable for MAYV transmission. A summary of the population living in potentially suitable MAYV transmission zones is presented in Table 2. Overall, we estimate that approximately 58.9 million people (95% CI: 21.4–100.4) in Central and South America live in areas that are potentially suitable for MAYV transmission. Countries with the greatest population living in suitable transmission zones include 46.2 million in Brazil (95% CI: 17.6–68.9), 3.5 million in Colombia (95% CI: 0.6–11.0), and 2.4 million in Panama (95% CI: 1.4–3.1). The majority of the Brazilian population living in suitable areas reside in the Amazon rainforest ecoregion. For our sensitivity analysis, we used lower (0.4) and higher (0.6) suitability values [69] to dichotomize the final map as suitable or unsuitable, and subsequently calculated the total population living in suitable transmission zones. Based on these alternate suitability values, the total population would be 77.2 million (95% CI: 35.7–120.6) and 49.8 million (95% CI: 16.5–91.3) for the 0.4 and 0.6 values, respectively.

**Table 2:** Total population living in areas potentially suitable for MAYV transmission (millions).

Country	Population in millions (95% CI)
Brazil	46.2 (17.6–68.9)
Colombia	3.5 (0.6–11.0)
Panama	2.4 (1.4–3.1)
Venezuela	2.2 (0.5–5.9)
Peru	1.3 (0.2–5.7)
Trinidad & Tobago	0.9 (0.6–1.0)
Ecuador	0.7 (0.0–2.5)
Bolivia	0.5 (0.1–1.0)
Suriname	0.5 (0.2–0.5)
Guyana	0.3 (0.1–0.4)
French Guiana	0.3 (0.1–0.3)
Overall	**58.9 (21.4–100.4)**

## Discussion

In this paper we present an ensemble BRT model of MAYV environmental suitability in Central and South America and an evidence consensus framework that integrates multiple information sources. Such suitability models can serve an important role in guiding arboviral surveillance (in humans and other hosts, including informing regional laboratory-based surveillance) and targeting vector control efforts. This is especially true in the case of MAYV given its nonspecific febrile presentation and the uncertainty surrounding its true distribution and non-human animal reservoirs. Our model provides important information regarding regions of Central and South America that are at highest risk of MAYV transmission allowing us to estimate the total population living in areas suitable for MAYV transmission. Furthermore, we are able to identify areas with high predicted MAYV suitability despite low or moderate evidence consensus of known transmission.

Our model predicted the distribution of MAYV with relatively high accuracy, identifying several regions of high environmental suitability. This included large areas of North and Central Brazil (e.g., the states of Mato Grosso, Pará, and Goiás), French Guiana, Trinidad & Tobago, and Northern Peru, all areas with well-documented evidence of MAYV transmission [70]. In addition, several regions with limited published evidence of MAYV transmission were also found to be highly suitable for transmission, including the majority of Guyana and Suriname. This finding highlights the utility of distribution models in identifying areas within countries that are particularly receptive to MAYV transmission that could be targeted for increased laboratory-based surveillance or vector control.

The wide predicted geographic distribution of MAYV underscores the need for increased surveillance and diagnostic capacity throughout Central and South America. Our findings suggest that MAYV may be underreported and that co-occurring arboviral epidemics (e.g., DENV or CHIKV) may obfuscate the true MAYV disease burden. This is especially true in Brazil, where ~43 million people reside in areas that are potentially suitable for MAYV transmission. An additional concern is the detection of MAYV in Haiti [71]. The discovery of MAYV in Haiti has prompted additional questions about its potential vectors and the possibility of urban transmission, due to the lack of *Haemagogus (Hag*.*) janthinomys* mosquitoes and non-human primates on the island. These questions require further entomological investigations in order to elucidate the vectorial capacity of urban mosquitoes and potential animal reservoirs other than non-human primates.

Another understudied aspect of the MAYV ecology is the impact of deforestation/land use change on transmission risk. This is an important consideration given the potential spread of MAYV in urban areas [13] and the demonstrated competence of *Aedes* mosquitoes in laboratory settings [14]. Epidemiological studies have demonstrated the link between residing or working in forested areas and MAYV risk [11, 12]. Environmental degradation and urbanization have led to increased contact between human populations and disease-carrying vectors, leading to elevated arbovirus transmission risk in general [72]. While this was not a specific focus of this study, we did account for the importance of land use/land cover in our analysis with the inclusion of EVI, evergreen forest, and urban land cover variables. While these variables contributed less to MAYV suitability model predictions compared to other climatic variables (e.g., rainfall), they still predicted MAYV transmission risk. Future studies of MAYV transmission risk could consider the specific impact of current and projected deforestation or land use change in order to predict future MAYV risk.

Our paper has several important limitations. First, there is significant heterogeneity of transmission within countries which may not be adequately captured by the evidence consensus scores. However, these country-level scores may help inform national-level approaches to surveillance and vector control, as well as help define transmission risks for international travel. In future work, evidence consensus scores may be generated for smaller geographic regions (e.g., first or second-level administrative divisions) as more data becomes available. Another limitation is related to the MAYV occurrence locations used as input data in the BRT environmental suitability model. Georeferenced reports of disease occurrence are subject to sampling bias related to the accessibility of certain locations, availability of laboratory infrastructure, or the presence of a robust surveillance system that is able to detect arbovirus occurrence. Therefore, the presence locations used in our model are likely subject to sampling bias and may not reflect the true distribution of MAYV. Following previously published modeling studies [62], we adjusted for the sampling bias in our dataset through the use of pseudoabsence points with a similar spatial bias as the presence points. However, the model may still be affected by sampling bias in the occurrence locations. Ideally, future MAYV distribution models will be updated when new occurrence locations are reported (as in Pigott et al. [73] updates to the Ebola suitability map), leading to a more accurate prediction of the true MAYV distribution.

Another limitation relates to the covariate set used to fit our model. Many aspects of the MAYV epidemiology remain poorly understood and current knowledge of MAYV ecology is limited. We included covariates that likely have a strong influence on MAYV transmission, including climate, landcover, and vegetation indices, which impact vector ecology and therefore transmission risk. However, MAYV transmission risk may be influenced by other unknown or understudied variables including socioeconomic factors and the presence of non-human reservoir hosts. Although several non-human primate species appear to be important MAYV reservoirs [9], we opted not to include primate distribution in our model due to uncertainty regarding their precise role in transmission cycles [27] in contrast to YFV [54]. As more research is conducted on MAYV ecology and additional animal reservoirs are identified, future MAYV distribution models should be updated to include primate distribution as a contributor to overall spillover risk. Our results here may assist in targeting and designing animal reservoir studies.

## Conclusion

In this study, we produced a high-resolution map of predicted MAYV environmental suitability as well as evidence consensus scores of known transmission for countries in the Americas. Although there is still much to learn about MAYV ecology and epidemiology, these results may help inform national-level transmission risk awareness and guide tailored, local disease surveillance and vector control efforts. Furthermore, these evidence consensus scores and environmental suitability maps can be updated as new MAYV occurrence data is reported and with additional variables in the future as the role of other drivers of MAYV transmission are clarified.

## Disclaimer

Material has been reviewed by the Walter Reed Army Institute of Research. There is no objection to its presentation and/or publication. The contents, views or opinions expressed in this publication are those of the authors and do not necessarily reflect official policy or position of Henry M. Jackson Foundation for the Advancement of Military Medicine, Inc., Uniformed Services University of the Health Sciences, Walter Reed Army Institute of Research, the Department of Defense (DoD), or Departments of the Army, Navy, or Air Force. Mention of trade names, commercial products, or organizations does not imply endorsement by the U.S. Government.

## Supporting information

S1 Fig The per-pixel standard deviation across the 100 sub-models is presented as a measure of the model’s uncertainty. The base map was sourced from Global Administrative Areas (GADM) version 4.0: https://gadm.org/download_country.html.
(TIF)

S2 Fig Schematic representation of the methods. (TIF)

S1 Table Evidence consensus score by country. (DOCX)

S2 Table Evidence consensus by state (Brazil). (DOCX)
