# Supplementary figures and images for: Understanding transmission risk and predicting environmental suitability for Mayaro Virus in Central and South America
Source: PLoS Negl Trop Dis. 2024 Jan 9;18(1):e0011859. doi: 10.1371/journal.pntd.0011859 (PMC10775973; doi:10.1371/journal.pntd.0011859)

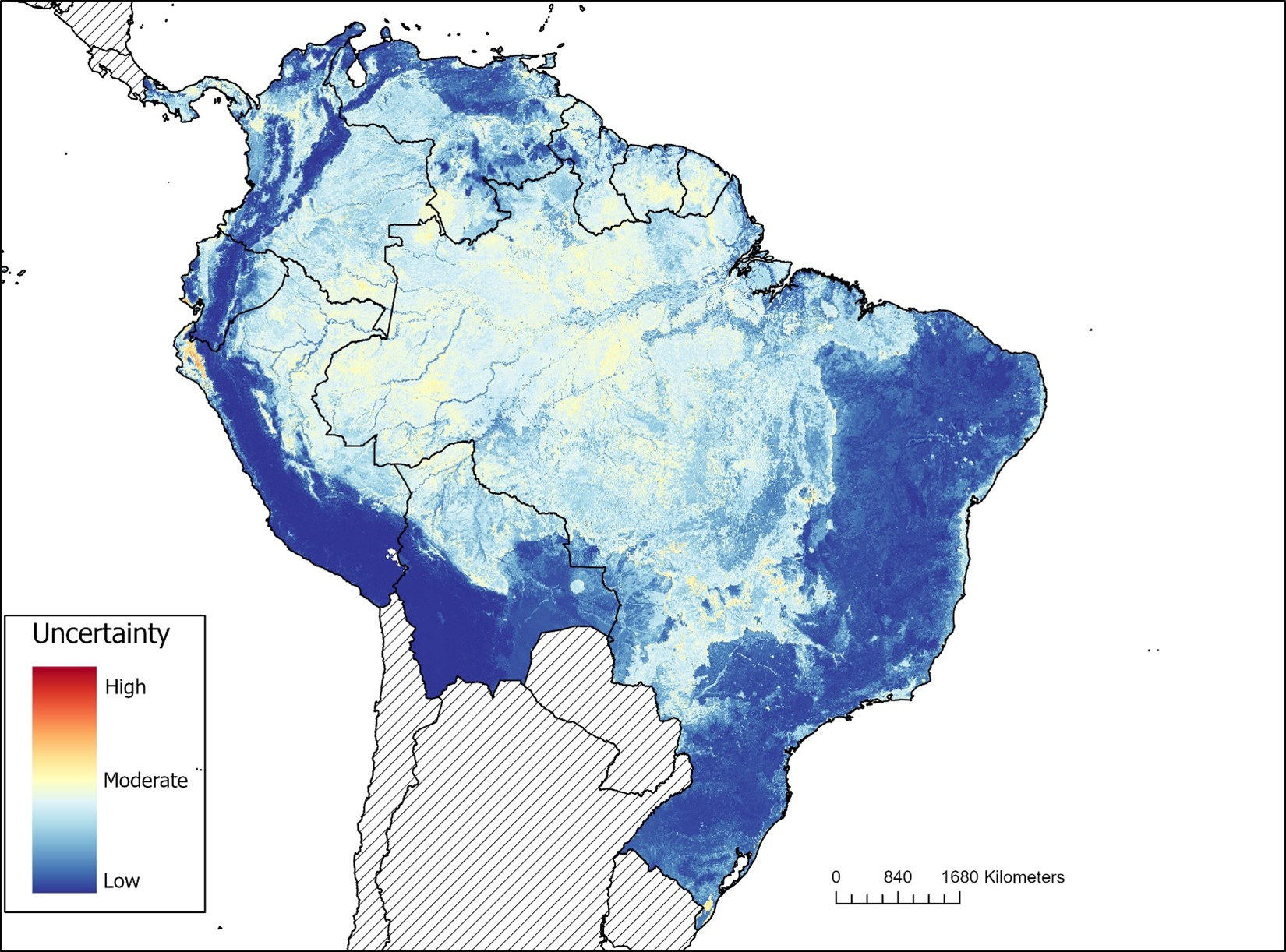

Supplement: S1 Fig — The base map was sourced from Global Administrative Areas (GADM) version 4.0: https://gadm.org/download_country.html. (TIF) [file pntd.0011859.s001.tif]

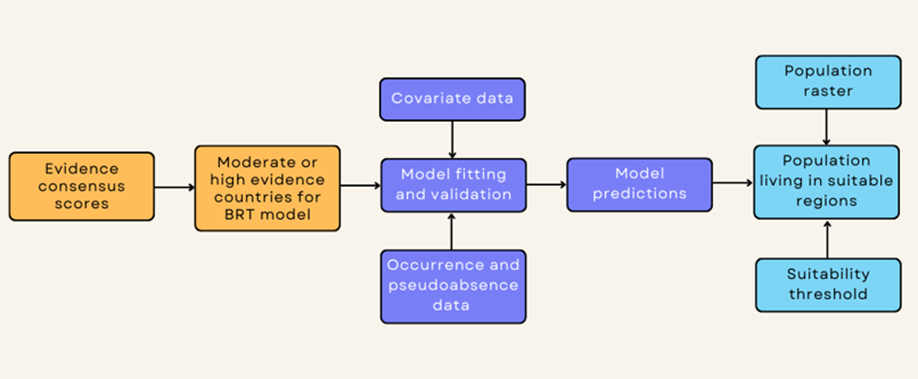

Supplement: S2 Fig — (TIF) [file pntd.0011859.s002.tif]
