# Supplementary material for: Understanding transmission risk and predicting environmental suitability for Mayaro Virus in Central and South America
Source: PLoS Negl Trop Dis. 2024 Jan 9;18(1):e0011859. doi: 10.1371/journal.pntd.0011859 (PMC10775973; doi:10.1371/journal.pntd.0011859)
Supplement: S1 Table — (DOCX) [file pntd.0011859.s003.docx]

## S1 Table: Evidence consensus score by country

| **Country** | **Health Org. Status** | **Date of Human Occurrence** | **Diagnostic Procedure** | **Outbreaks and Clinical Cases** | **Health Expenditure and Adjacency** | **Animal Data** | **Arthropod Data** | **Total Score** |
| --- | --- | --- | --- | --- | --- | --- | --- | --- |
| **Brazil** | **1** | **3** | **3** | **6** | **N/A** | **3** | **3** | **19- Very high** |
|  | GIDEON only | Most recent occurrence: 2021 [1] | RT-PCR [2] | 26 clinical cases in 2017-18 [2] |  | Detected in horses in 2016 [3] | Detected in mosquitoes 2018-19 [4] |  |
| **Venezuela** | **3** | **3** | **3** | **6** | **N/A** | **1** | **0** | **16- Very high** |
|  | GIDEON and PAHO alert [5] | Most recent occurrence: 2016 [6] | Isolation [6] | 77 clinical cases during outbreak in 2010 [7] |  | Detected in sentinel hamster in 1999 [8] | None detected |  |
| **Peru** | **1** | **3** | **3** | **6** | **N/A** | **2** | **0** | **15- High** |
|  | GIDEON only | Most recent occurrence: 2019 [9] | RT-PCR and isolation [10] | 86 clinical cases in 2016 [11] |  | Detected in various animals in 2008 [12] | None detected |  |
| **French Guiana** | **3** | **3** | **3** | **3** | **N/A** | **1** | **0** | **13- High** |
|  | GIDEON and PAHO alert [13] | Most recent occurrence: 2020 [13] | RT-PCR [13] | 13 clinical cases in 2020 [13] |  | Detected in various animals in 1994-95 [14] | None detected |  |
| **Trinidad & Tobago** | **1** | **3** | **3** | **3** | **N/A** | **0** | **3** | **13- High** |
|  | GIDEON only | Most recent occurrence: 2014 [15] | RT-PCR [15] | 9 clinical cases in 2014 [15] |  | None detected | Identified by metatranscriptomics in 2018 [16] |  |
| **Bolivia** | **1** | **2** | **3** | **5** | **N/A** | **0** | **0** | **11- High** |
|  | GIDEON only | Most recent occurrence: 2007 [17] | RT-PCR and isolation [17] | 23 clinical cases from 2000-07 [17] |  | None detected | None detected |  |
| **Colombia** | **1** | **1** | **3** | **0** | **4** | **1** | **1** | **11- High** |
|  | GIDEON only | Most recent occurrence: 1966 [18] | NT [19] | No clinical cases | Per capita CHE was high; adjacent to Brazil, Venezuela, Peru | Detected in primates in 1957 [19] | Isolated from mosquito in 1958-60 [20] |  |
| **Haiti** | **1** | **3** | **3** | **3** | **N/A** | **0** | **0** | **10- Moderate** |
|  | GIDEON only | Most recent occurrence:  2015 [21] | RT-PCR [21] | 5 clinical cases 2014-15 [21] |  | None detected | None detected |  |
| **Suriname** | **1** | **1** | **3** | **0** | **5** | **0** | **0** | **10- Moderate** |
|  | GIDEON only | Most recent occurrence: 1964 [22] | Isolation [23] | No clinical cases | Per capita CHE was medium; adjacent to Brazil and French Guiana | None detected | None detected |  |
| **Ecuador** | **1** | **3** | **3** | **2** | **N/A** | **0** | **0** | **9- Moderate** |
|  | GIDEON only | Most recent occurrence: 2019 [24] | Isolation, RT-PCR, or IgM seroconversion (not specified) [17] | 1 clinical case 2000-07 [17] |  | None detected | None detected |  |
| **Guyana** | **1** | **1** | **2** | **0** | **5** | **0** | **0** | **9- Moderate** |
|  | GIDEON only | Most recent occurrence: 1955 [25] | Serology (not specified) [25] | No clinical cases | Per capita CHE was medium; adjacent to Brazil and Venezuela | None detected | None detected |  |
| **Panama** | **1** | **3** | **3** | **0** | **0** | **1** | **1** | **9- Moderate** |
|  | GIDEON only | Most recent occurrence: 2017 [26] | NT [26] | No clinical cases | No neighbors | Detected in various animals in 1974-76 [27] | Isolated from mosquito in 1972-79 [28] |  |
| **Mexico** | **1** | **2** | **2** | **0** | **0** | **0** | **0** | **5- Low** |
|  | GIDEON only | Most recent occurrence: 2001 [29] | IgM ELISA [29] | No clinical cases | No neighbors | None detected | None detected |  |
| **Paraguay** | **0** | **0** | **0** | **0** | **5** | **0** | **0** | **5- Low** |
|  | Neither | No cases | No cases | No clinical cases | Per capita CHE was medium; adjacent to Brazil and Bolivia | None detected | None detected |  |
| **Argentina** | **0** | **0** | **0** | **0** | **4** | **0** | **0** | **4- Low** |
|  | Neither | No cases | No cases | No clinical cases | Per capita CHE was high; adjacent to Brazil and Bolivia | None detected | None detected |  |
| **Chile** | **0** | **0** | **0** | **0** | **4** | **0** | **0** | **4- Low** |
|  | Neither | No cases | No cases | No clinical cases | Per capita CHE was high; adjacent to Bolivia and Peru | None detected | None detected |  |
| **Costa Rica** | **0** | **1** | **2** | **0** | **0** | **0** | **0** | **3- Low** |
|  | Neither | Most recent occurrence: 1968 [30] | HI test [30] | No clinical cases | No neighbors | None detected | None detected |  |
| **Dominican Republic** | **0** | **0** | **0** | **0** | **2** | **0** | **0** | **2- Low** |
|  | Neither | No cases | No cases | No clinical cases | Per capita CHE was medium; adjacent to Haiti | None detected | None detected |  |
| **Uruguay** | **0** | **0** | **0** | **0** | **1** | **0** | **0** | **1- Low** |
|  | Neither | No cases | No cases | No clinical cases | Per capita CHE was high; adjacent to Brazil | None detected | None detected |  |
| **Belize** | **0** | **0** | **0** | **0** | **0** | **0** | **0** | **0- Low** |
|  | Neither | No cases | No cases | No clinical cases | No neighbors | None detected | None detected |  |
| **Guatemala** | **0** | **0** | **0** | **0** | **0** | **0** | **0** | **0- Low** |
|  | Neither | No cases | No cases | No clinical cases | No neighbors | None detected | None detected |  |
| **Honduras** | **0** | **0** | **0** | **0** | **0** | **0** | **0** | **0- Low** |
|  | Neither | No cases | No cases | No clinical cases | No neighbors | None detected | None detected |  |
| **Nicaragua** | **0** | **0** | **0** | **0** | **0** | **0** | **0** | **0- Low** |
|  | Neither | No cases | No cases | No clinical cases | No neighbors | None detected | None detected |  |
| **El Salvador** | **0** | **0** | **0** | **0** | **0** | **0** | **0** | **0- Low** |
|  | Neither | No cases | No cases | No clinical cases | No neighbors | None detected | None detected |  |

HI: hemagglutination inhibition; NT: neutralization test; RT-PCR: Reverse transcription polymerase chain reaction; CHE: Current health expenditure

References

1. Outbreak News. Brazil: 8 Mayaro virus cases reported in Pará in 2021 2022. Available from: <https://outbreaknewstoday.com/brazil-8-mayaro-virus-cases-reported-in-para-in-2021/>.

2. de Paula Silveira-Lacerda E, Herlinger AL, Tanuri A, Rezza G, Anunciação CE, Ribeiro JP, et al. Molecular epidemiological investigation of Mayaro virus in febrile patients from Goiania City, 2017-2018. Infect Genet Evol. 2021:104981. doi: <https://doi.org/10.1016/j.meegid.2021.104981>.

3. Gomes FA, Jansen AM, Machado RZ, Jesus Pena HF, Fumagalli MJ, Silva A, et al. Serological evidence of arboviruses and coccidia infecting horses in the Amazonian region of Brazil. PloS One. 2019;14(12):e0225895. doi: <https://doi.org/10.1371/journal.pone.0225895>.

4. de Curcio JS, Salem-Izacc SM, Pereira Neto LM, Nunes EB, Anunciação CE, Silveira-Lacerda EP. Detection of Mayaro virus in Aedes aegypti mosquitoes circulating in Goiânia-Goiás-Brazil. Microbes Infect. 2022;24(4):104948. doi: <https://doi.org/10.1016/j.micinf.2022.104948>.

5. Pan American Health Organization. Epidemiological Alert, Outbreak of Mayaro Fever in the Americas (7 June 2010) 2010. Available from: <https://iris.paho.org/handle/10665.2/50840>.

6. Blohm GM, Marquez-Colmenarez MC, Lednicky JA, Bonny TS, Mavian C, Salemi M, et al. Isolation of Mayaro virus from a Venezuelan patient with febrile illness, arthralgias, and rash: further evidence of regional strain circulation and possible long-term endemicity. Am J Trop Med Hyg. 2019;101(6):1219-25. doi: <https://doi.org/10.4269/ajtmh.19-0357>.

7. Auguste AJ, Liria J, Forrester NL, Giambalvo D, Moncada M, Long KC, et al. Evolutionary and Ecological Characterization of Mayaro Virus Strains Isolated during an Outbreak, Venezuela, 2010. Emerging Infectious Diseases. 2015;21(10):1742-50. doi: <https://doi.org/10.3201/eid2110.141660>.

8. Medina G, Garzaro DJ, Barrios M, Auguste AJ, Weaver SC, Pujol FH. Genetic diversity of Venezuelan alphaviruses and circulation of a Venezuelan equine encephalitis virus subtype IAB strain during an interepizootic period. Am J Trop Med Hyg. 2015;93(1):7-10. doi: <https://doi.org/10.4269/ajtmh.14-0543>.

9. Gobierno Regional de Cusco - Direccion Regional de Salud Cusco. Boletín de Vigilancia en Salud Pública. Direccion Ejecutiva de Intelligencia Sanitaria,2019.

10. de la Cruz CH, Martínez SLA, Failoc-Rojas VE, Aguilar-Gamboa FR. Momento de considerar otras arbovirosis luego del virus Mayaro. Rev Cubana Med Gen Integr. 2019;35.

11. Aguilar-Luis MA, Del Valle-Mendoza J, Sandoval I, Silva-Caso W, Mazulis F, Carrillo-Ng H, et al. A silent public health threat: emergence of Mayaro virus and co-infection with dengue in Peru. BMC Res Notes. 2021;14(1):29. doi: <https://doi.org/10.1186/s13104-021-05444-8>.

12. Perez JG, Carrera JP, Serrano E, Pitti Y, Maguina JL, Mentaberre G, et al. Serologic evidence of zoonotic alphaviruses in humans from an indigenous community in the Peruvian Amazon. Am J Trop Med Hyg. 2019;101(6):1212-18. doi: <https://doi.org/10.4269/ajtmh.18-0850>.

13. World Health Organization. Mayaro virus disease - French Guiana, France 2020 [cited 2020 November ]. Available from: <https://www.who.int/csr/don/25-october-2020-mayaro-fever-french-guiana-france/en/>.

14. de Thoisy B, Gardon J, Salas RA, Morvan J, Kazanji M. Mayaro virus in wild mammals, French Guiana. Emerg Infect Dis. 2003;9(10):1326-9. doi: 10.3201/eid0910.030161.

15. Gonzalez-Escobar G, Churaman C, Rampersad C, Singh R, Nathaniel S. Mayaro virus detection in patients from rural and urban areas in Trinidad and Tobago during the chikungunya and Zika virus outbreaks. Pathog Glob Health. 2021:1-9. doi: <https://doi.org/10.1080/20477724.2021.1878445>.

16. Ali R, Jayaraj J, Mohammed A, Chinnaraja C, Carrington CVF, Severson DW, et al. Characterization of the virome associated with Haemagogus mosquitoes in Trinidad, West Indies. Sci Rep. 2021;11(1):16584. doi: <https://doi.org/10.1038/s41598-021-95842-6>.

17. Forshey BM, Guevara C, Laguna-Torres VA, Cespedes M, Vargas J, Gianella A, et al. Arboviral etiologies of acute febrile illnesses in western South America, 2000-2007. PLoS Negl Trop Dis. 2010;4(8):e787. doi: 10.1371/journal.pntd.0000787.

18. Prías-Landínez DE, Bernal-Cúbides C, de Torres SV, Romero-León M. Encuesta serologica de virus transmitidos por artropodos. Bol Oficina Sanit Panama. 1970.

19. Groot H. Estudios sobre virus transmitidos por artropodos en Colombia. Rev Acad Colomb Cienc. 1964;12(46):191-217. doi: <https://doi.org/10.18257/raccefyn.565>.

20. Groot H, Morales A, Vidales H. Virus isolations from forest mosquitoes in San Vicente de Chucuri, Colombia. Am J Trop Med Hyg. 1961;10:397-402. doi: <https://doi.org/10.4269/ajtmh.1961.10.397>.

21. Blohm G, Elbadry MA, Mavian C, Stephenson C, Loeb J, White S, et al. Mayaro as a Caribbean traveler: evidence for multiple introductions and transmission of the virus into Haiti. International journal of infectious diseases : IJID : official publication of the International Society for Infectious Diseases. 2019;87:151-3. doi: 10.1016/j.ijid.2019.07.031.

22. Karbaat J. Arbovirus infections in Dutch military personnel stationed in Surinam. Trop Geogr Med. 1964;17(2):187-89.

23. Metselaar D. Isolation of arboviruses of group A and group C in Surinam. Trop Geogr Med. 1966;18(2):137-42.

24. Pan American Health Organization / World Health Organization. Epidemiological alert: Mayaro fever Washington, D.C.: PAHO/WHO2019 [updated May 1, 2019]. Available from: <https://www.paho.org/en/documents/epidemiological-alert-mayaro-fever-1-may-2019>.

25. Downs WG, Anderson CR. Distribution of immunity to Mayaro virus infection in the West Indies. West Indian Med J. 1958;7(3):190-94.

26. Carrera JP, Cucunubá ZM, Neira K, Lambert B, Pittí Y, Liscano J, et al. Endemic and epidemic human alphavirus infections in eastern Panama: an analysis of population-based cross-sectional surveys. Am J Trop Med Hyg. 2020;103:2429-37. doi: <https://doi.org/10.4269/ajtmh.20-0408>.

27. Seymour C, Peralta PH, Montgomery GG. Serologic evidence of natural togavirus infections in Panamanian sloths and other vertebrates. Am J Trop Med Hyg. 1983;32(4):854-61. doi: <https://doi.org/10.4269/ajtmh.1983.32.854>.

28. Galindo P, Adames A, Peralta P, Johnson C, Read R. Impacto de la hidroeléctrica de Bayano en la transmisión de arbovirus. Rev Med Pan. 1983;8:89-134.

29. Navarrete-Espinosa J, Gomez-Dantes H. Arbovirus causales de fiebre hemorrágica en pacientes del Instituto Mexicano del Seguro Social. Revista medica del Instituto Mexicano del Seguro Social. 2006;44(4):347-53.

30. Fuentes LG, Mora JA. Encuesta serologica sobre arbovirus en Costa Rica. Rev Latinoam Microbiol. 1971;13(1):25-8.
