# Supplementary material for: Understanding transmission risk and predicting environmental suitability for Mayaro Virus in Central and South America
Source: PLoS Negl Trop Dis. 2024 Jan 9;18(1):e0011859. doi: 10.1371/journal.pntd.0011859 (PMC10775973; doi:10.1371/journal.pntd.0011859)
Supplement: S2 Table — (DOCX) [file pntd.0011859.s004.docx]

## S2 Table. Evidence consensus by state (Brazil)

| **Country** | **Health Org. Status^1^** | **Date of Human Occurrence** | **Diagnostic Procedure** | **Outbreaks and Clinical Cases** | **Health Expenditure and Adjacency** | **Animal Data** | **Arthropod Data** | **Total Score** |
| --- | --- | --- | --- | --- | --- | --- | --- | --- |
| **Mato Grosso** | 1 | 3 | 3 | 6 | N/A | 0 | 3 | **16- Very high** |
|  | GIDEON only | Most recent occurrence: 2017 [1] | RT-PCR [2] | 68 positives by RT-PCR from 2011-17 [2-4] |  | No cases | Mosquito pools positive by RT-PCR and isolation in 2018 [5] |  |
| **Goiás** | **1** | **3** | **3** | **6** | **N/A** | **0** | **3** | **16- Very high** |
|  | GIDEON only | Most recent occurrence: 2017-18 [6, 7] | RT-PCR [6, 7] | 104 positives by RT-PCR from 2016-17 [6, 7] |  | No cases | Mosquitoes positive by RT-PCR in 2018-2019 [8] |  |
| **Pará** | **1** | **3** | **3** | **3** | **N/A** | **2** | **2** | **14- High** |
|  | GIDEON only | Most recent occurrence: 2016 [9] | RT-PCR [9] | 4 positives by RT-PCR in 2016 [9] |  | Infected animals in 2009 [10] | Isolated from mosquitoes in 2008 [11] |  |
| **Bahia** | **1** | **1** | **2** | **0** | **5** | **3** | **2** | **14- High** |
|  | GIDEON only | Most recent occurrence: 1984 [12] | Serology [12] | No outbreak or clinical cases | Per capita CHE was medium; adjacent to Piauí and Goiás | Infected animal from 2012-17 [13] | One pool positive by RT-PCR from 2009-14 [14] |  |
| **Roraima** | **1** | **3** | **3** | **3** | **N/A** | **3** | **0** | **13- High** |
|  | GIDEON only | Most recent occurrence: 2012 [15] | RT-PCR [15] | 7 positives by RT-PCR in 2012 [15] |  | Infected animals in 2016 [16] | No cases |  |
| **Piauí** | **1** | **3** | **3** | **3** | **N/A** | **2** | **0** | **12- High** |
|  | GIDEON only | Most recent occurrence: 2016-17 [17] | RT-PCR [17] | One positive by RT-PCR from 2016-17 [17] |  | Infected animal from 2008-10 [18] | No cases |  |
| **Mato Grosso do Sul** | **1** | **2** | **3** | **2** | **N/A** | **3** | **0** | **11- High** |
|  | GIDEON only | Most recent occurrence: 2000 [19] | Viral culture [19] | One isolate in 2000 [19] |  | Infected animals from 2012-14 [20] | No cases |  |
| **Amazonas** | **1** | **3** | **3** | **3** | **N/A** | **0** | **0** | **10- Moderate** |
|  | GIDEON only | Most recent occurrence: 2016 | RT-PCR [21] | 13 positives by RT-PCR from 2014-16 [21] |  | No cases | No cases |  |
| **Maranhão** | **1** | **3** | **3** | **3** | **N/A** | **0** | **0** | **10- Moderate** |
|  | GIDEON only | Most recent occurrence: 2016-18 [22] | RT-PCR [22] | One positive by RT-PCR from 2016-18 [22] |  | No cases | No cases |  |
| **Tocantins** | **1** | **3** | **3** | **3** | **N/A** | **0** | **0** | **10- Moderate** |
|  | GIDEON only | Most recent occurrence: 2017 [23] | RT-PCR [23] | 6 positives by RT-PCR in 2017 [23] |  | No cases | No cases |  |
| **Acre** | **1** | **2** | **2** | **0** | **5** | **0** | **0** | **10- Moderate** |
|  | GIDEON only | Most recent occurrence: 2004 [24] | Serology [24] | No outbreak or clinical cases | Per capita CHE was medium; adjacent to Amazonas and Peru | No cases | No cases |  |
| **Amapá** | **1** | **1** | **2** | **0** | **5** | **0** | **0** | **9- Moderate** |
|  | GIDEON only | Most recent occurrence: 1995 [25] | Serology [25] | No outbreak or clinical cases | Per capita CHE was medium; adjacent to French Guiana and Para | No cases | No cases |  |
| **São Paulo** | **1** | **3** | **2** | **0** | **2** | **0** | **0** | **8- Moderate** |
|  | GIDEON only | Most recent occurrence: 2017 [26] | ELISA [26] | No clinical cases | Per capita CHE was medium; adjacent to Mato Grosso do Sul | No cases | No cases |  |
| **Paraíba** | **1** | **1** | **3** | **0** | **0** | **2** | **0** | **7- Moderate** |
|  | GIDEON only | Most recent occurrence: 1964 [27] | Plaque reduction NT [27] | No outbreak or clinical cases | No neighbors | Infected animal from 2008-10 [18, 28] | No cases |  |
| **Rondônia** | **1** | **0** | **0** | **0** | **5** | **1** | **0** | **7- Moderate** |
|  | GIDEON only | No cases | No cases | No outbreak or clinical cases | Per capita CHE was medium; adjacent to Bolivia, Amazonas, Mato Grosso | Infected animals in 1987-88 [29] | No cases |  |
| **Rio Grande do Sul** | **1** | **0** | **0** | **0** | **0** | **2** | **3** | **6- Moderate** |
|  | GIDEON only | No cases | No cases | No clinical cases | No neighbors | Infected animal in 2002 [30] | Isolated from mosquitoes 2011 |  |
| **Minas Gerais** | **1** | **0** | **0** | **0** | **5** | **0** | **0** | **6- Moderate** |
|  | GIDEON only | No cases | No cases | No clinical cases | Per capita CHE was medium; adjacent to Goias and Mato Grosso do Sul | No cases | No cases |  |
| **Rio de Janeiro** | **1** | **3** | **1** | **0** | **0** | **0** | **0** | **5- Low** |
|  | GIDEON only | Most recent occurrence: 2019 [31] | Not specified [31] | No clinical cases | No neighbors | No cases | No cases |  |
| **Pernambuco** | **1** | **0** | **0** | **0** | **2** | **2** | **0** | **5- Low** |
|  | GIDEON only | No cases | No cases | No outbreak or clinical cases | Per capita CHE was medium; adjacent to Piaui | Infected animal from 2008-10 [18] | No cases |  |
| **Alagoas** | **1** | **0** | **0** | **0** | **0** | **2** | **0** | **3- Low** |
|  | GIDEON only | No cases | No cases | No outbreak or clinical cases | No neighbors | Infected animal from 2008-10 [18] | No cases |  |
| **Rio Grande do Norte** | **1** | **0** | **0** | **0** | **0** | **2** | **0** | **3- Low** |
|  | GIDEON only | No cases | No cases | No clinical cases | No neighbors | Infected animal from 2008-10 [18] | No cases |  |
| **Paraná** | **1** | **0** | **0** | **0** | **2** | **0** | **0** | **3- Low** |
|  | GIDEON only | No cases | No cases | No outbreak or clinical cases | Per capita CHE was medium; adjacent to Mato Grosso do Sul | No cases | No cases |  |
| **Ceará** | **1** | **0** | **0** | **0** | **2** | **0** | **0** | **3- Low** |
|  | GIDEON only | No cases | No cases | No clinical cases | Per capita CHE was medium; adjacent to Piaui | No cases | No cases |  |
| **Espírito Santo** | **1** | **0** | **0** | **0** | **0** | **0** | **0** | **1- Low** |
|  | GIDEON only | No cases | No cases | No clinical cases | No neighbors | No cases | No cases |  |
| **Santa Catarina** | **1** | **0** | **0** | **0** | **0** | **0** | **0** | **1- Low** |
|  | GIDEON only | No cases | No cases | No clinical cases | No neighbors | No cases | No cases |  |
| **Sergipe** | **1** | **0** | **0** | **0** | **0** | **0** | **0** | **1- Low** |
|  | GIDEON only | No cases | No cases | No clinical cases | No neighbors | No cases | No cases |  |

^1^ Due to lack of data availability at the ADM1 level, the overall Health Organization Status score for the country of Brazil was applied to each state.

References

1. Fumagalli MJ, de Souza WM, Romeiro MF, de Souza Costa MC, Slhessarenko RD, Figueiredo LTM. Development of an Enzyme-Linked Immunosorbent Assay To Detect Antibodies Targeting Recombinant Envelope Protein 2 of Mayaro Virus. Journal of clinical microbiology. 2019;57(5). doi: <https://doi.org/10.1128/jcm.01892-18>.

2. de Souza Costa MC, Siqueira Maia LM, Costa de Souza V, Gonzaga AM, Correa de Azevedo V, Ramos Martins L, et al. Arbovirus investigation in patients from Mato Grosso during Zika and Chikungunya virus introdution in Brazil, 2015-2016. Acta Trop. 2019;190:395-402. doi: <https://doi.org/10.1016/j.actatropica.2018.12.019>.

3. Pilatti M, de Almeida Paiva LEC, de Carli BNR, de Souza Costa MC, Zuchi N, Slhessarenko RD, et al. Perfil clínico-epidemiológico dos pacientes infectados com o vírus Mayaro (MAYV) em Mato Grosso. TCC-Biomedicina. 2018.

4. Zuchi N, Heinen LB, Santos MA, Pereira FC, Slhessarenko RD. Molecular detection of Mayaro virus during a dengue outbreak in the state of Mato Grosso, Central-West Brazil. Memorias do Instituto Oswaldo Cruz. 2014;109(6):820-3. doi: <https://doi.org/10.1590/0074-0276140108>.

5. da Silva Neves NA, da Silva Ferreira R, Morais DO, Pavon JAR, de Pinho JB, Slhessarenko RD. Chikungunya, Zika, Mayaro, and Equine Encephalitis virus detection in adult Culicinae from South Central Mato Grosso, Brazil, during the rainy season of 2018. Brazilian journal of microbiology : [publication of the Brazilian Society for Microbiology]. 2022;53(1):63-70. doi: <https://doi.org/10.1007/s42770-021-00646-5>.

6. Correa JF. Avaliação sorológica e molecular de pacientes com suspeita de arboviroses em uma unidade municipal de saúde de Goiânia-Goiás. 2020. [Thesis]: Universidade Federal de Goiás; 2020.

7. de Paula Silveira-Lacerda E, Herlinger AL, Tanuri A, Rezza G, Anunciação CE, Ribeiro JP, et al. Molecular epidemiological investigation of Mayaro virus in febrile patients from Goiania City, 2017-2018. Infect Genet Evol. 2021:104981. doi: <https://doi.org/10.1016/j.meegid.2021.104981>.

8. de Curcio JS, Salem-Izacc SM, Pereira Neto LM, Nunes EB, Anunciação CE, Silveira-Lacerda EP. Detection of Mayaro virus in Aedes aegypti mosquitoes circulating in Goiânia-Goiás-Brazil. Microbes Infect. 2022;24(4):104948. doi: <https://doi.org/10.1016/j.micinf.2022.104948>.

9. Saatkamp CJ, Rodrigues LRR, Pereira AMN, Coelho JA, Marques RGB, Souza VC, et al. Mayaro virus detection in the western region of Pará state, Brazil. Rev Soc Bras Med Trop. 2021;54:e0055-2020. doi: <https://doi.org/10.1590/0037-8682-0055-2020>.

10. Casseb AdR, Brito TC, Silva MRMd, Chiang JO, Martins LC, Silva SPd, et al. Prevalence of antibodies to equine alphaviruses in the State of Pará, Brazil. Arq Inst Biol. 2016;83. doi: <https://doi.org/10.1590/1808-1657000202014>.

11. Azevedo RS, Silva EV, Carvalho VL, Rodrigues SG, Neto JPN, Monteiro HA, et al. Mayaro fever virus, Brazilian amazon. Emerg Infect Dis. 2009;15(11):1830. doi: <https://doi.org/10.3201/eid1511.090461>.

12. Tavares-Neto J, Rosa APATd, Vasconcelos PFC, Costa JML, Rosa JFSTd, Marsden PD. Pesquisa de anticorpos para arbovírus no soro de residentes no povoado de Corte de Pedra, Valença, Bahia. Memorias do Instituto Oswaldo Cruz. 1986;81(4):351-8. doi: <https://doi.org/10.1590/S0074-02761986000400001>.

13. Moreira-Soto A, Carneiro ID, Fischer C, Feldmann M, Kummerer BM, Silva NS, et al. Limited Evidence for Infection of Urban and Peri-urban Nonhuman Primates with Zika and Chikungunya Viruses in Brazil. mSphere. 2018;3(1). doi: <https://doi.org/10.1128/mSphere.00523-17>.

14. Catenacci LS. Abordagem one health para vigilância de arbovirus na Mata Atlântica do sul da Bahia, Brasil. [Dissertation]. Ananindeua: Instituto Evandro Chagas; 2017.

15. Meneses CAR. Identificação de arbovírus, Mayaro e Oropouche em amostras com dengue negativo NS1 no estado de Roraima, no ano de 2012 [Thesis]: Universidade Federal de Roraima; 2013.

16. Gomes FA, Jansen AM, Machado RZ, Jesus Pena HF, Fumagalli MJ, Silva A, et al. Serological evidence of arboviruses and coccidia infecting horses in the Amazonian region of Brazil. PloS One. 2019;14(12):e0225895. doi: <https://doi.org/10.1371/journal.pone.0225895>.

17. Barros ELT. Caracterização molecular de chikungunya virus e investigação dos arbovírus dengue virus e Mayaro virus no estado do Piauí [Thesis]: Universidade Federal do Piauí; 2018.

18. Laroque PO, Valença-Montenegro MM, Ferreira DRA, Chiang JO, Cordeiro MT, Vasconcelos PFC, et al. Levantamento soroepidemiológico para arbovírus em macaco-prego-galego (Cebus flavius) de vida livre no estado da Paraíba e em macaco-prego (Cebus libidinosus) de cativeiro do nordeste do Brasil. Pesq Vet Bras. 2014;34:462-8.

19. Coimbra TL, Santos CL, Suzuki A, Petrella SM, Bisordi I, Nagamori AH, et al. Mayaro virus: imported cases of human infection in Sao Paulo State, Brazil. Rev Inst Med Trop Sao Paulo. 2007;49(4):221-4. doi: <https://doi.org/10.1590/s0036-46652007000400005>.

20. Paulo MB, Renato A, Rocha; TCd, C.G.; E, Silva; MANd, Walfrido KS, et al. Serosurvey of Arbovirus in Free-Living Non-Human Primates (Sapajus Spp.) in Brazil. Int J Environ Anal Chem. 2015;2(4). doi: <https://doi.org/10.4172/2380-2391.1000155>.

21. da Silva MS. Estudo de casos suspeitos de dengue negativos no teste sorológico para detecção do antígeno NS1: falha no diagnóstico ou emergência de outras arboviroses? [Thesis]: Universidade Federal do Amazonas; 2017.

22. Nunes JPP, de Melo BO, Monteiro SG, Almeida VSS, Monteiro AS, Cosme LMSS, et al. One-Step reverse transcriptase PCR for detection of arboviruses in serum samples of patients assisted in Basic health Units in the State of Maranhão, Brazil. Braz J Dev. 2019;5. doi: <https://doi.org/https://doi.org/10.34117/bjdv5n9-203>.

23. Dos Santos Souza Marinho R, Duro RLS, Bellini Caldeira D, Galinskas J, Oliveira Mota MT, Hunter J, et al. Re-emergence of mayaro virus and coinfection with chikungunya during an outbreak in the state of Tocantins/Brazil. BMC Res Notes. 2022;15(1):271. doi: <https://doi.org/10.1186/s13104-022-06153-6>.

24. da Silva-Nunes M. The Acre Project: the epidemiology of malaria and arthropod-borne virus infections in a rural Amazonian population. Cad Saúde Pública. 2006;22(6):1325-34.

25. Souto RNP, Souto RCC. Sorologia para arbovírus em população humana da região da Reserva Extrativista do Cajari, Amapá, Brasil. Biota Amazônia. 2012;2(2):8-14. doi: <http://dx.doi.org/10.18561/2179-5746/biotaamazonia.v2n2p8-14>.

26. Romeiro MF, Fumagalli MJ, Dos Anjos AB, Figueiredo LTM. Serological evidence of Mayaro virus infection in blood donors from São Carlos, São Paulo, Brazil. Trans R Soc Trop Med Hyg. 2020;114(9):686-9. doi: <https://doi.org/10.1093/trstmh/traa016>.

27. Niederman JC, Henderson JR, Opton EM, Black FL, Skvrnova K. A nationwide serum survey of Brazilian military recruits, 1964. II. Antibody patterns with arboviruses, polioviruses, measles and mumps. Am J Epidemiol. 1967;86(2):319-29. doi: <https://doi.org/10.1093/oxfordjournals.aje.a120742>.

28. Araujo FAA, Andrade MA, Jayme VS, Santos AL, Roman APM, Ramos DG, et al. Anticorpos antialfavírus detectados em equinos durante diferentes epizootias de encefalite equina, Paraíba, 2009. Rev Bras Ciênc Vet. 2012;19(1):80-5. doi: <https://doi.org/10.4322/rbcv.2014.086>.

29. Degallier N, Travassos da Rosa AP, Vasconcelos PFC, Hervé JP, Sa Filho GC, Travassos da Rosa JFS, et al. Modifications of arbovirus transmission in relation to construction of dams in Brazilian Amazonia. Ciência e Cultura. 1992;44:124-35.

30. Araujo FAA, Wada MY, da Silva EV, Cavalcante GC, Magalhaes VS, de Andrade Filho GV, et al. Primeiro inquérito sorológico em aves migratórias e nativas do Parque Nacional da Lagoa do Peixe/RS para detecção do vírus do Nilo Ocidental. Boletim Eletrônico Epidemiológico (Secretaria de Vigilância em Saúde). In: Saúde MdSSdVe, editor. Boletim Eletrônico Epidemiologico2003. p. 2-8.

31. Ziegler MF. Mechanism that triggers the inflammatory process by Mayaro virus is discovered: São Paulo Research Foundation; 2019 [cited 2021]. Available from: <https://agencia.fapesp.br/mechanism-that-triggers-the-inflammatory-process-during-infection-by-mayaro-virus-is-discovered/32066/>.
